# A pilot newborn screening program for Mucopolysaccharidosis type I in Taiwan

**DOI:** 10.1186/1750-1172-8-147

**Published:** 2013-09-22

**Authors:** Shuan-Pei Lin, Hsiang-Yu Lin, Tuen-Jen Wang, Chia-Ying Chang, Chia-Hui Lin, Sung-Fa Huang, Chia-Chen Tsai, Hsuan-Liang Liu, Joan Keutzer, Chih-Kuang Chuang

**Affiliations:** 1 Division of Genetics and Metabolism, Department of Medical Research, Mackay Memorial Hospital, Taipei, Taiwan; 2 Department of Pediatrics, Mackay Memorial Hospital, Taipei, Taiwan; 3 Department of Laboratory Medicine, Mackay Memorial Hospital, Taipei, Taiwan; 4 Institute of Biotechnology, National Taipei University of Technology, Taipei, Taiwan; 5 College of Medicine, Fu-Jen Catholic University, New Taipei City, Taiwan; 6 Department of Early Childhood Care and Education, Mackay Junior College of Medicine, Nursing and Management,, Taipei, Taiwan; 7 Department of Medicine, Mackay Medical College, New Taipei City, Taiwan; 8 Department of Biochemistry, School of Medical Laboratory Science and Biotechnology, Taipei Medical University, Taipei, Taiwan; 9 Department of Pediatrics, Mackay Memorial Hospital, Hsinchu, Taiwan; 10 Institute of Clinical Medicine, National Yang-Ming University, Taipei, Taiwan; 11 Scientific and Medical Affairs, Genzyme Corporation, Cambridge, MA, USA

**Keywords:** Mucopolysaccharidosis type I, α-L-iduronidase, Dried blood spots, Newborn screening

## Abstract

**Background:**

Mucopolysaccharidosis type I (MPS I) is a genetic disease caused by the deficiency of α-L-iduronidase (IDUA) activity. MPS I is classified into three clinical phenotypes called Hurler, Scheie, and Hurler-Scheie syndromes according to their clinical severity. Treatments for MPS I are available. Better outcomes are associated with early treatment, which suggests a need for newborn screening for MPS I. The goal of this study was to determine whether measuring IDUA activity in dried blood on filter paper was effective in newborn screening for MPS I.

**Methods:**

We conducted a newborn screening pilot program for MPS I from October 01, 2008 to April 30, 2013. Screening involved measuring IDUA activity in dried blood spots from 35,285 newborns using a fluorometric assay.

**Results:**

Of the 35,285 newborns screened, 19 did not pass the tests and had been noticed for a recall examination. After completing further recheck process, 3 were recalled again for leukocyte IDUA enzyme activity testing. Two of the three had deficient leukocyte IDUA activity. Molecular DNA analyses confirmed the diagnosis of MPS I in these two newborns.

**Conclusions:**

It is feasible to use the IDUA enzyme assay for newborn screening. The incidence of MPS I in Taiwan estimated from this study is about 1/17,643.

## Introduction

The mucopolysaccharidoses (MPS) are a group of inherited diseases known as lysosomal storage disorders. Each MPS is caused by the deficiency of a different enzyme that catalyzes the stepwise degradation of glycosaminoglycans (GAG; mucopolysaccharides) in lysosomes. Mucopolysaccharidosis type I (MPS I) is inherited in an autosomal-recessive fashion and caused by deficiency of α-L-iduronidase (IDUA; EC 3.2.1.76) activity and progressive accumulation of its substrates, partially degraded dermatan and heparin sulfate, in lysosomes. The accumulation of dermatan and heparan sulfate in lysosomes trigger changes in cellular metabolism that lead to tissue and organ damage and the signs and symptoms of MPS I. Patients with MPS I can have coarse facial features, developmental delay and decline, gibbus deformity, hepatosplenomegaly, cardiac valve disease, umbilical and inguinal hernias, joint restriction, upper airway complications, sleep apnea, recurrent otitis media, and premature death. MPS I is classified into three clinical phenotypes called Hurler, Scheie, and Hurler-Scheie syndromes according to their clinical severity [1-3].

Treatment for MPS I involves supportive and symptom-based treatment and disease-specific treatments that address the underlying cause of the disease. Disease-specific treatments to replace IDUA activity include laronidase enzyme replacement therapy for the non-neurologic manifestations of MPS I patients [4-12] and bone marrow and hematopoietic stem cell transplantation for Hurler syndrome patients [13-16]. de Ru et al. reported that enzyme replacement therapy (ERT) can result in good clearance of GAGs from many tissues and can significantly ameliorate several symptoms [12]. However, some of the clinical symptoms of MPS I are irreversible and bone marrow and hematopoietic stem cell transplantation must precede developmental decline [14]. Thus, early diagnosis is crucial and positively related to the response of MPS I patients to disease-specific therapies. Data from siblings who began treatment at different ages suggest that initiation of laronidase treatment in infancy, before the development of significant disease manifestations, may improve outcome with respect to coarse facial features, musculoskeletal disease, cardiac valve disease [17] and brain MRI abnormalities [18]. These results suggest that early diagnosis of MPS I through newborn screening may improve the prognosis of patients with MPS I.

MPS I patients were 5.3% of the MPS patients diagnosed in Taiwan from 1984 to 2004. A Taiwanese MPS I birth prevalence of 0.11 per 100,000 live births was estimated using diagnostic frequency data, population data, and a review of all MPS I patient’s medical records [19]. This estimated birth prevalence is lower than expected, but may reflect undiagnosed and misdiagnosed MPS I patients. We conducted a pilot MPS I newborn screening program in Taiwan from October 1, 2008 through April 30, 2013. The objectives of the study were to determine whether measuring IDUA activity in dried blood on filter paper was effective in newborn screening for MPS I and to determine the birth prevalence of MPS I in Taiwan.

## Materials and methods

### Dried blood spot sample preparation

Dried-blood spot (DBS) samples were prepared using the National Committee for Clinical Laboratory Standards protocol (NCCLS standard LA4-A4), “Blood Collection on Filter Paper for Neonatal Screening Program” [20,21]. Blood samples were collected by heel puncture on the third day of life. Drops of blood were spotted directly on filter paper (ToYo PKU No.545, Japan), and dried at least 4 hours at room temperature. DBS samples were stored at 4°C in zipper-lock plastic bags. IDUA activity was measured in two 3.2-mm-diameter disks from each DBS. From October 1, 2008 to April 30, 2013, a total of 35,285 samples from newborns were collected from our hospital. DBS samples from two patients with a confirmed diagnosis of MPS I (before and after ERT) and 4 MPS I obligate carriers were also included in this study. All samples were collected following written informed consent.

### Chemicals

All chemicals were obtained from Sigma Co., and the substrate, 4-methylumbelliferyl-α-L-iduronide, was purchased from Toronto Research Chemicals in Canada.

### Control DBS filter papers

Blood collected in EDTA from normal volunteers and pooled was used to make a normal control (C1, n = 20) and an inactivated control (C2, n = 20). Inactivated control DBS were placed in a 50°C oven for 96 hours to degrade IDUA activity. These control dried blood spots on filter paper (DBFPs) were used for assay quality control.

### IDUA enzyme assay

A modification of the method published by Chamoles et al. was used [22,23]. Elution buffer contains 50 mmol/L formate buffer (pH 2.8) and 3.57 μmol/L d-saccharic acid-1, 4-lactone. A reaction mixture contained the elute solution and the substrate (4-methylumbelliferyl-α-L-iduronide) in a 2:1 ratio (v/v). One 3.2 mm disc, which contains approximate 3.55 μL of blood was punched from each DBS using a Wallac DBS puncher (Perkin Elmer, Inc., USA), and placed into 96-well black microtiter plate (F 96, Nunc, Denmark). Thirty μL of reaction mixture (two parts elution buffer and 1 part substrate) was added to each well. The plate was covered well with foil to avoid influences from light, air, and humidity. The plate was incubated at 37°C in an orbital shaker at 250 rpm (Medclub Scientific CB-2201) for 20 h. One-hundred and fifty μL of glycine-carbonate buffer (stop solution) was added to each well and the plate was kept at room temperature for 30 minutes to stop the reaction. The fluorescence of the enzyme product, 4-methylumbelliferone (4-MU), was measured by using a microplate Fluorometer (Victor 2D® Neonatal Fluorometer, Perkin Elmer) with excitation 360 nm and emission 450 nm. The filter paper did not need to be removed during the analysis. The fluorescence readings were corrected for blanks, and the results were compared with the fluorescence from a 4-MU calibration curve. IDUA enzymatic activities were expressed as micromoles of substrate hydrolyzed per liter of blood per 20 h.

### Calibration curve

A calibration curve was made fresh each time the test was performed. A 50 mM 4-MU stock solution was prepared in DMSO. A 0.02 μM working standard solution was first prepared in deionized water, and diluted serially to generate seven calibrators at concentrations of 0.02, 0.008, 0.0032, 0.00128, 0.000512, 0.0002048 μM. Water was used as a blank. The regression analysis (*r*^2^) of the calibration curve (fluorescence reading *vs.* concentrations of 4-MU calibrations) was excellent (*r*^2^ = 0.991).

## Results

In the study, forty samples were run and calculated for within-run and between-run precisions (Coefficient of Variance; CV%). All the 40 samples were run in 6 consecutive replicates for within-run analysis, and a duplicate analysis of each sample (n = 40) was performed on 6 different days sequentially. The within-run and between-run precisions of DBFP fluorescence enzyme assay were 9.35% and 11.99%, respectively. In order to ensure the stability and the accuracy of fluorescence enzyme assay in DBFP, the within-run and between-run precisions of control DBFPs (C1 and C2) were also evaluated, which were 7.11% and 8.57% for C1, as well as 9.16% and 8.70% for C2, respectively. The overall IDUA activities in average of C1 and C2 were 54.48 (± 4.51) and 31.55 (± 2.92) μmol/L blood*20 h.

**Figure 1:**
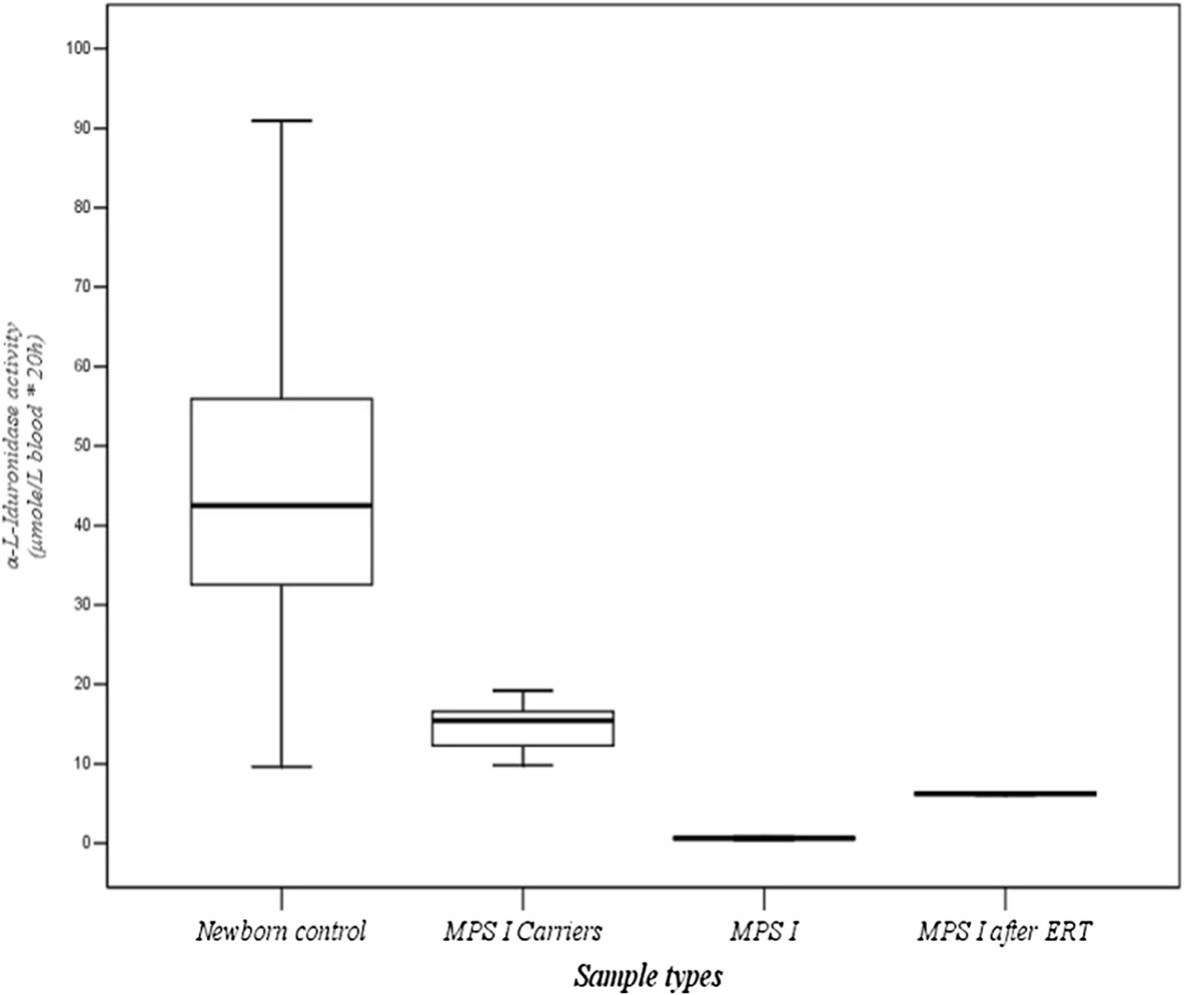
The reference values of IDUA activities in newborn control (n = 35,285), MPS I carriers (n = 4), confirmed MPS I patients (n = 2), and the same patients received ERT.

**Figure 2:**
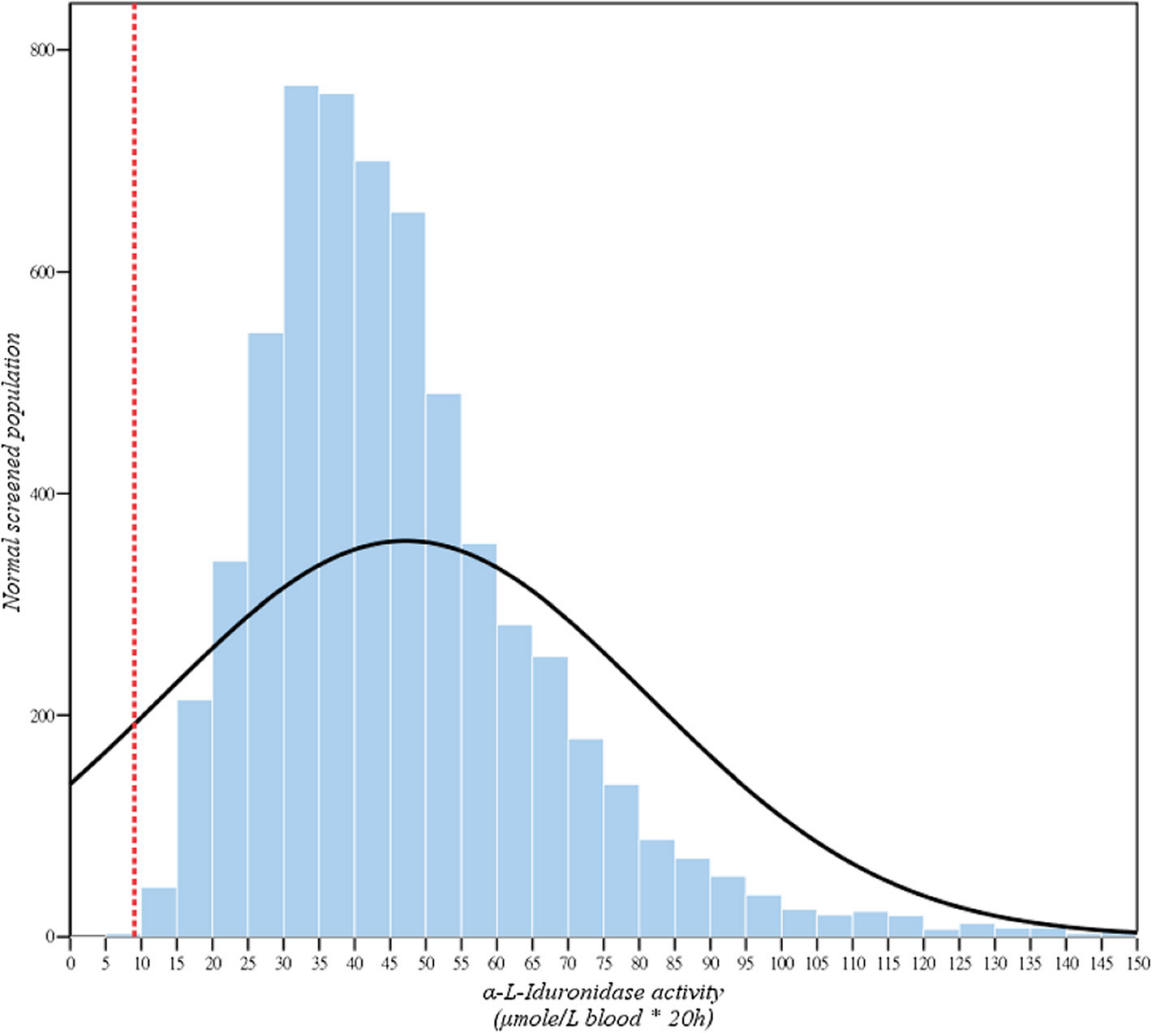
The sampling to determine the reference values of IDUA activities in newborn control showed a normal distribution (Kolmogorov-Smimov Z = 7.703).

The reference values of IDUA activities using DBFP fluorescence enzyme assay were 9.03 - 69.52 μmol/L blood*20 h (Mean ± SD = 39.58 ± 15.12; n = 2173) (Figure 1), and the samples showed a normal distribution (Kolmogorov-Smimov Z = 7.703) analyzed with the SPSS for Windows 11.0 program (SPSS Inc., Chicago, IL, USA) (Figure 2). For the confirmed MPS I patients before ERT (n = 2), the IDUA activities were both less than 5% of the normal population, and ranged from 0.29 to 0.95 μmol/L blood*20 h. The IDUA activities obtained from the same patients who have received ERT by weekly infusion (n = 2) showed significantly elevated about ten-fold increases, ranged from 5.53 to 6.77 μmol/L blood*20 h. The IDUA activities of the carriers who are the parents of the confirmed MPS I patients (n = 4) were slightly reduced in concentrations when comparing with the normal values, ranged from 9.40 to 19.82 μmol/L blood*20 h.

In this MPS I newborn screening project, a total of 35,285 newborns were analyzed. In those, 58 newborns had IDUA activity less than 9.03 μmol/L blood*20 h at the first screening test. Most of them (n = 39) were exclusive and their data showed normal instead after a second rechecked analysis with the original DBS samples. The other 19 infants did not pass the tests and had been noticed for a recall examination. All 19 neonates (recalled rate = 0.054%) had completed the further rechecked process, and only three infants still had IDUA activities in DBFP lower than 9.03 μmol/L blood*20 h. In order to confirm the diagnosis of those subjects, a series of tests including urine GAG quantification (DMB/CRE ratio), urinary GAG two-dimensional electrophoresis (2-D EP), leukocyte IDUA enzymatic assay, and molecular DNA analysis had been arranged. All the methods described previously [24,25] or from literatures reported elsewhere [26]. The results of the above confirmative methods showed that 2 infants had marked deficiency of IDUA activities were typical of MPS I patients (less than 5% of the reference values, 6.8-37.0 μmol/g protein/hr), and another that showed definitely reduction in IDUA activity might be a carrier (3.5 μmol/g protein/hr). The urinary DMB ratios of the 3 infants were all normal comparing to the reference values of newborn (44.6 ± 23.7 mg/mmol creatinine). The 2-D EP results showed a distinct dermatan sulfate but a faint heparan sulfate pattern in the 2 affected infants and another (carrier suspected) was normal.

The molecular DNA analysis was demonstrated on the 2 highly suspicious MPS I infants and their parents based on the methods reported by Sun et al. [27] and Wang et al. [28]. In the study, the PCR primer sequences and PCR conditions were modified and listed in Table 1. PCR products were purified and sequenced using ABI Prism 3730 DNA sequencer (Applied Biosystems). All amplified fragments flanking exons were analyzed to identify variations. Mutations were further verified by restriction analysis of PCR products. Two point mutations were identified in the infant A confined as c.303 G > A (R105Q) that inherited from father/ c.484 G > A (R162K) that inherited from mother. For the infant B, the point mutations were also identified and confined as c.344 G > T (D119Y) inherited from father/ c.99 T > G (H33Q) from mother. All sequence changes were confirmed by restriction enzyme testing on a second PCR product, and the restriction enzyme tests for 4 known mutations in both MPS I patients were listed in Table 2. Molecular genetic testing of IDUA to determine carrier status was performed to both parents of infant C who was highly suspected to be an MPS I carrier.

**Table 1:** PCR primer sequences, amplicon length and PCR reaction temperature

**Exon**	**Primer sequence**	**Amplicon length (bp)**	**Annealing temperature (°C)**
1	F:5′-CCGCAGTCCCGAGCAC-3′	277	60
	R:5′-GCTCCGGTCTCTGAAGCTCT-3′		
2	F: 5′-CCCTCGTCTTACTGCTGCTG-3′	401	60
	R: 5′TCCCATCTGTGCCTCTGTAA-3′		
3,4	F: 5′-CATACCAGGCCTTCATAGGG-3′	708	58
	R: 5′CCAACCTATCCCTTGTCACC-3′		
5	F: 5′-CATCACCTTGCACCCTCC-3′	273	60
	R: 5′-CGTCTACACCTGCCCTGG-3′		
6	F: 5′-CCGCTCATCCCCAGGGCAGGTGTA-3′	301	60
	R: 5′-ACAGCGGCTGAGGGCGCAGAACAC-3′		
7	F: 5′-CATCTCCCTCCACAGGAAGGTG-3′	500	60
	R: 5′-GGTAGCTCAGGAAGGCATTGTC-3′		
8	F: 5′-TTCCTCCCGAGACGGGACAGGCGA-3′	437	60
	R: 5′-CTCCCCTTGGTGAAGGAGTC-3′		
9	F: 5′-TGGGGACTCCTTCACCAAGGGGAG-3′	370	60
	R: 5′-CAGAGCCCCAGCGGGGCCAGAGAC-3′		
10	F: 5′-ATCTACGCGAGCGACGAC-3′	470	60
	R: 5′-GGTCCTCAGGGTTCTCCAG-3′		
11	F: 5′-GTGTGGGTGGGAGGTGGAGCGGTG-3′	302	60
	R: 5′-AGGGAAGGGCTGTGAGGCGTCGG-3′		
12	F: 5′-GCTTTTGCTGGTGCACGTGT-3′	295	60
	R: 5′-AAGTGGCCCGAGTGACCGCAT-3′		
13,14	F: 5′-CCTAGGGGACATGAGATGGA-3′	814	58
	R: 5′-CGGGGTTTACCCTTGGAG-3′		

**Table 2:** Restriction enzyme test for 4 known mutations in MPS I patients

**Patients**	**Mutation**	**Primers**	**Restriction enzyme**	**Fragment size (bp)**
Infant A	R105Q	F: 5′-CCTTCTGCAGGGGGTCCACTGT-3′	Bsr GI	N: 286
Infant A-father	(exon3)	R: 5′-CAAACCCTGGAACACAGGAG-3′		M: 286 + 262 + 24
Infant A	R162K	F: 5′-ACCCACCTGGACGGGTACCGG-3′	Apo I	N: 357
Infant A-mother	(exon4)	R: 5′-TGCGCTCGCCCACCGATGAAT-3′		M: 357 + 339 + 18
Infant B	D119Y	F: 5′-CATACCAGGCCTTCATAGGG-3′	Bsr GI	N: 251
Infant B-father	(exon3)	R: 5′-TGGTTCTCCCTGAGAATGT-3′		M: 251 + 234 + 17
Infant B	H33Q	F: 5′-GAGGCCCCGCACCTGCTGCA-3′		N: 174
Infant B-mother	(exon1)	R: 5′-GCTCCGGTCTCTGAAGCTCT-3′	Pst I	M: 174 + 154 + 20

## Discussion and conclusion

The reported birth incidence of MPS I in Taiwan (0.11 per 100,000 live births) is equivalent to the prevalence rate in UK published by Murphy et al. [29], and about one tenth of the prevalence reported by Moore et al. [30]. The overall incidence of MPS is estimated to be 1 in 10,000 ~ 22,500 births in Europe [1-3,31], and MPS I is by far the relatively rare type. Nelson et al. had published and estimated an incidence of 1 in 76,000 for Hurler syndrome in Northern Ireland, which was proposed to be higher than previously recorded from Lowry and Renwick in 1971 [32,33]. Murphy et al. reported that the birth incidence was 1 in 26,206 with a carrier frequency of 1 in 81 [29], which is nearly three-fold higher than that reported by Nelson. A more recently published report by Scott, et al. showed a higher frequency of MPS I (1 in 35,700) in Washington State, USA [34] which was 3-fold greater than the prevalence estimated from clinical studies [31,35]. Based on the results in this study, the actual incidence of MPS I in Taiwan is about 1 in 17,643 births, and indicates that there are approximately 10 to 12 new patients to be born each year in Taiwan. For early detection purpose, making early diagnosis is extremely valuable, and thus giving them early therapy can help saving lives and preventing brain damage or other health problems.

**Figure 3:**
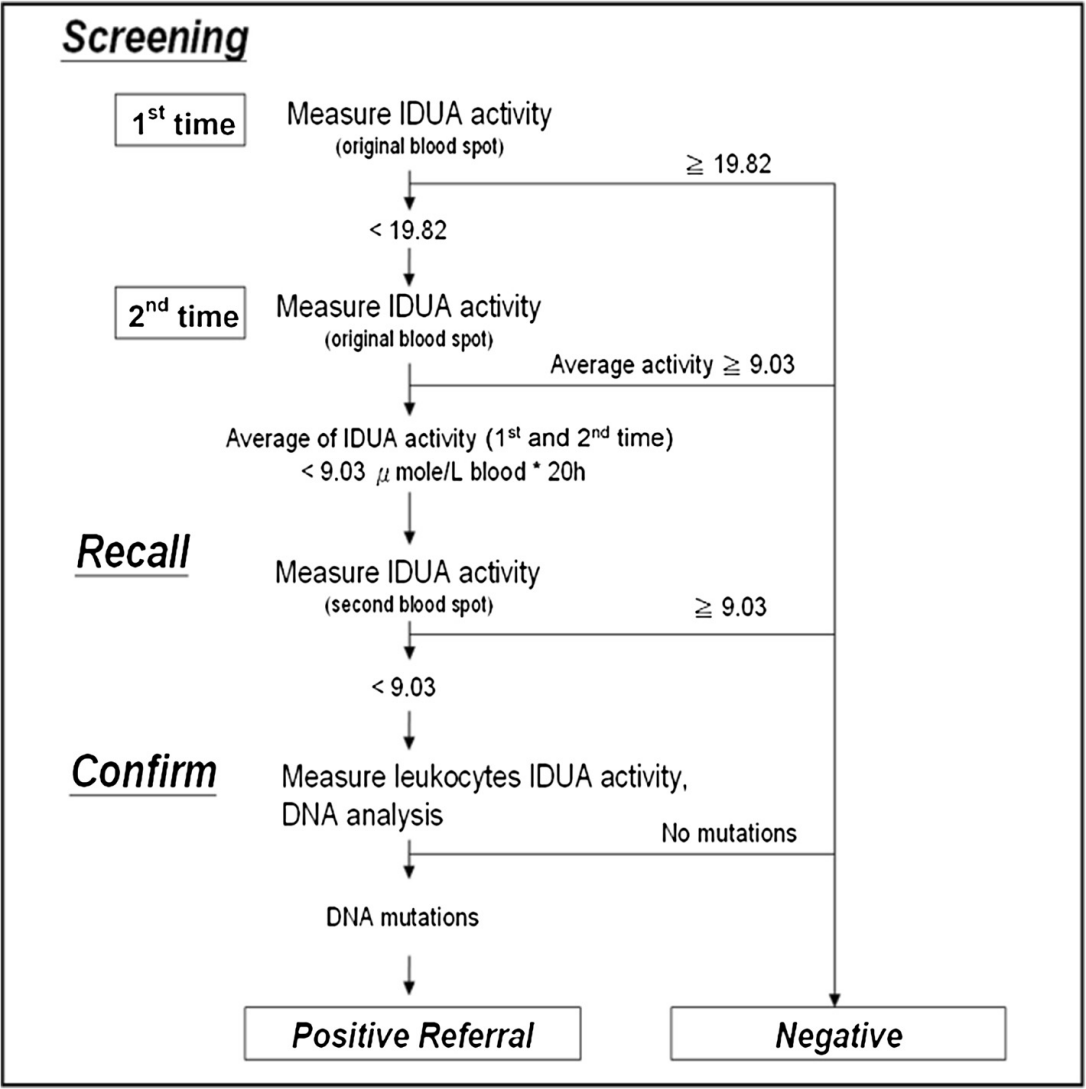
A diagnostic algorithm for MPS I newborn screening test.

In the past few years, many screening methods have been proposed for MPS I newborn screening purposes, including immunochemistry assay [36], tandem mass spectrometry method [37-39], and fluorescent enzymatic assay [22,23]. In those, the developments of multiplex assay by using tandem mass spectrometry newborn screening for detectable congenital metabolic diseases, particularly the lysosomal storage diseases (LSD) is thought to be the main stream worldwide in the future. However, those tandem mass assays still have many obstacles to overcome, such as the high-priced instrumentation, unstable substrate and commercially unavailable in part, time-consuming procedures, complexity, and high total cost. The immunochemistry assay using DBFP is another issue commonly used for LSD newborn screening purposes; however, the specificity and the purity of an antibody may affect the ability between antigen and antibody conjugation. Additionally, a false-negative result may be produced due to dysfunction of the detectable antigen (enzyme). In this study, the determination of IDUA activity in DBFP using fluorescence enzyme assay is a secure method for MPS I diagnosis. The DBS fluorescence enzyme assay has many advantages including simple performance, cost effectiveness, high sensitivity and high specificity, high throughput, and limited false positive detection. A diagnostic algorithm for MPS I is provided in Figure 3. The first or a further second testing on the original DBFP sample is accomplished that may rule out the false positive samples from the highly suspicious cases including the carriers. The cut-off points of IDUA activities in DBFP by using fluorescence enzymatic assay were 19.82 and 9.03 μmol/L blood*20 h in the first and second time tests, respectively. The parents of highly suspicious infants will receive a recall notice for a second DBFP sample collection if the result of DBS fluorescence enzyme assay is less than the cut-off point, 9.03 μmol/L blood*20 h. The confirmative examinations such as leukocyte enzymatic assay and molecular DNA analysis will be performed while the second rechecked DBS fluorescence enzyme assay failed.

The IDUA activity detected by using DBS fluorescence enzyme assay is adapted for the initial diagnosis of MPS I. However, it is extremely important to guarantee the quality of DBFP samples, including collection, transport, and storage. According to the results published by Chamoles et al., there were no significant changes in enzyme activity of DBFP samples after storage for 21 days at 4°C or −20°C; in contrast, the IDUA activity will significantly decrease while the DBFP samples are stored at room temperature for the same period [21-23]. In our study, the collection, transport, and storage of DBFP samples all followed the requirements of the National Committee for Clinical Laboratory Standards protocol (NCCLS standard LA4-A4), and the quality of the DBFP was ensured.

In summary, the fluorescent enzymatic assay has the potential to be adopted for newborn screening of MPS I. The method is reliable, sensitive, validated, easy to perform, and cost-effective in measuring IDUA activity in DBFP when comparing with the tandem mass spectrometry newborn screening method. The fluorescent enzymatic assay does not require an expensive instrumentation and trained staff, and the cost per assay is about two thirds of the tandem mass spectrometry method. The incidence of MPS I in this study is about 1/17,643 that might be overestimated due to the small sample size. In order to screen out more suspicious subjects actively and gauge prevalence accurately, we will continue and extend the sample numbers cooperated with other newborn screening centers in Taiwan.

## Competing interest

The authors declare that they have no competing interests.

## Authors’ contributions

SPL and HYL performed acquisition, statistical analysis and interpretation of data, and drafting of the manuscript. CKC participated in design of the study, interpretation of the data and helped to draft the manuscript. TJW, CHL, SFH, CCT, HLL, and JK performed biochemical analyses and revised the manuscript. CYC was responsible for patient screening. All authors read and accepted the manuscript.
